# Purification and characterization of neutral protease from *Aspergillus oryzae* Y1 isolated from naturally fermented broad beans

**DOI:** 10.1186/s13568-018-0611-6

**Published:** 2018-06-12

**Authors:** Xiao-lin Ao, Xi Yu, Ding-tao Wu, Chao Li, Tong Zhang, Shu-liang Liu, Shu-juan Chen, Li He, Kang Zhou, Li-kou Zou

**Affiliations:** Sichuan Agricultural University, Xinkang Road 46, Yaan, 625014 Sichuan China

**Keywords:** *Aspergillus oryzae*, Neutral protease, Screening and purification, Enzymatic properties

## Abstract

The strain Y1, with a notably high production of neutral protease, was isolated from naturally fermented broad beans and subsequently identified as *Aspergillus oryzae*, through the analysis of its morphology characteristics and 18S rDNA sequence. Naturally fermented broad beans are the main raw material in Sichuan broad-bean sauce. The neutral protease from *Aspergillus oryzae* Y1 was purified using ammonium sulphate precipitation and DEAE-Sepharose Fast Flow chromatography, which resulted in a 10.0-fold increase in the specific activity (2264.3 U/mg) and a recovery rate of 21%. The estimated molecular mass of the purified protease was approximately 45 kDa. The optimal pH and temperature of the purified protease were 7.0 and 55 °C, respectively. The heat resistance of the purified protease was significantly higher than the commercial protease. The effect of metal ions on the activity of the purified protease approximated that of commercial neutral protease. Furthermore, the maximum hydrolysis rate (*V*_max_) and apparent Michaelis–Menten constant (*K*_m_) values of the purified protease were 256.4103 μg/mL min and 20.0769 mg/mL, respectively. The purified protease had a higher affinity for the substrate than the commercial neutral protease. All the results suggest that this neutral protease exhibits the potential for application in industry due to its good resistance to high temperatures and wide range of acids and bases.

## Introduction

Proteases may be classified as either acidic protease, neutral protease or alkaline protease, according to their optimum pH reactions. Proteases, especially the neutral protease, make up the largest proportion of industrial enzymes around the world (Kasana et al. 2011). Neutral proteases are widely applied in the food (Tavano 2013; Yuzuki et al. 2015), feed (Zhang et al. 2014), pharmaceutical (Umeadi et al. 2008) and leather (Asker et al. 2013) industries because of their distinct benefits, including a mild catalysis process, low pollution level and high yield. In the food industry, the neutral protease is generally used for debittering soy sauce (Machida et al. 2005) and brewing beer (Wang et al. 2013). However, the neutral protease is generally poor in thermal stability and is easily deactivated, which limits its application in some industries that require high temperatures. Neutral proteases are produced primarily by microorganisms, animals and plants. Those produced by microorganisms are most commonly applied in industry, because of their strong hydrolytic ability and wide adaptability to catalytic reaction conditions, which make them more adaptable to downstream processing than those proteases obtained from plants or animals (Sandhya et al. 2005; Gupta et al. 2002). Furthermore, neutral fungal proteases present a level of higher peptidase activity compared to other commercial enzymatic preparations (Guerard et al. 2002). The main characteristic of this enzyme is its affinity toward hydrophobic amino acids, which is advantageous for its use as a debittering agent (Sumantha et al. 2005).

Numerous recent studies on fungal proteases have revealed them to be acidic or alkaline rather than neutral proteases; widely reported examples include the acid protease from *Aspergillus oryzae* HG76 (Li et al. 2014), *A. flavus* (Franco et al. 2017) and *A. foetidus* (Souza et al. 2015), with alkaline protease noted in *Aspergillus* sp. UCP 1276 (Ferreira et al. 2016), *A. terreus* (Biaggio et al. 2016), and *Rhizopus oryzae* (Mushtaq et al. 2015). Currently, the important focus for industrial application is to find a neutral protease derived from fungi that offers good thermal stability, acid–alkali resistance and a high affinity to protein.

Broad-bean sauce has been a popular traditional condiment in China for more than 300 years. It is produced commercially through a series of processes using mainly fermented broad beans. *Aspergillus* spp., especially *A. oryzae*, are the microorganisms predominantly utilized during the fermentation process of broad beans (Vishwanatha et al. 2009). They produce a diverse array of enzymes, such as protease (Murthy and Kusumoto 2015), amylase (Zhang et al. 2017), phytase (Sapna and Singh 2017) and cellulase (Takashima et al. 1998). Among these, the neutral protease is the most important and widely used enzyme. Therefore, in this study, in order to obtain a neutral protease that is resistant to high temperatures, strains with a high production of neutral protease were screened from broad beans naturally fermented at high temperatures and, thereafter, the neutral protease was further purified and characterized.

## Materials and methods

### Reagents, medium and strains

Commercial neutral protease, with high purity levels and a strong ability to hydrolyze protein, was acquired from Beijing Solarbio Science & Technology Company (Beijing, China), which conforms to the Food Chemical Codex (FCC) and the food grade enzymes specification standards of light industry (QB/T1803-93). The fungal DNA kit was purchased from OMEGA Engineering (Connecticut, America); DEAE-Sepharose Fast Flow was obtained from Beijing Ruida Henhui Science & Technology Development Company (Beijing, China); and the Coomassie brilliant blue Kit was from Nanjing Jiancheng Bioengineering Institute (Nanjing, China). Polyethylene glycol 2000-0 (PEG-20M) was obtained from Chengdu Jinshan Chemical Reagent Company (Chengdu, China). Tris, Folin phenol, and Triton X-100 were purchased from Beijing Solarbio Science & Technology Company (Beijing, China). Rose Bengal medium was obtained from Beijing Aobox Biotechnology Company (Beijing, China). All the other chemicals were analytical grade.

The fermented broad beans were collected under high temperature in summer from an open-air natural fermentation workshop, where the average temperature of the fermentation pool reaches 55 °C.

### Isolation and identification of strains

The strains from the fermented broad beans were isolated for use in this study using a Rose Bengal medium. The isolated strains were inoculated onto the casein medium (pH 7.0) at 35 °C for 48 h. The strain with the highest activity of proteinase was subsequently identified, according to the ratio of the hydrolytic zone diameter to the colony diameter.

The screened strain was inoculated into a Rose Bengal medium and then cultivated at 35 °C for 48 h. The characteristics of the fungal colony, such as shape, color, size and protuberance, were recorded and the cellular morphology was observed.

The genomic DNA of the strain was extracted by means of a silica-gel pillar fungal genomic DNA extraction kit. The 18S rDNA gene of the isolate was amplified by PCR using the primers EF-3 (5′-GGAAGGG (G/A) TGTATTTATTAG-3′) and EF-4 (5′-TCCT (A/C) TAAATGACCAAGTTTG-3′). Amplified PCR products were analyzed by means of agarose gel (1%, w/v) electrophoresis, and purified using the PCR purification kit. The purified products were sent to Sangon Biotech for DNA sequence analysis. The 18S rDNA sequence data was deposited in the NCBI with the accession number MF 374341. The phylogenetic tree was constructed using MEGA5.1 via a comparison with the 18S rDNA sequences of similar such microorganisms collected from the GenBank.

### Extraction of crude enzyme

The isolated strain Y1, with the high production of protease, was inoculated 10^7^ CFU/g into a bran medium consisting of 10 g bran and 10 mL distilled water. It was subsequently incubated at 35 °C for 48 h, before 100 mL buffer (50 mM Tris–HCl, pH 7.0) was mixed into the medium at 40 °C for 1 h for crude enzyme extraction. The crude extract was then separated by centrifugation at 4000*g* for 20 min, and the supernatant was retained for further analysis, in which the enzyme activity and content of protease were determined.

### Protease activity

Protease activity was determined according to the method described by Xiang et al. (2016). Briefly, after digestion of the azocasein, obtained from a casein chromophore containing a dinitrogenated arylamine, the protease activity was quantified based on the released peptides, and monitored by absorbance at 660 nm. One unit of protease activity was defined as the amount of 1 μg tyrosine from the casein, hydrolyzed per min at 40 °C under assay conditions.

### Purification of protease

#### Ammonium sulfate precipitation

The neutral protease was concentrated by adding ammonium sulphate (20–90%) at 4 °C, followed by overnight incubation. The sample was centrifuged at 4000*g* for 20 min at 4 °C. The enzyme activity and protein content of ammonium sulfate precipitation were determined. The precipitates were collected and then dialyzed with a Millipore 8–14 kDa dialysis bag against a 50 mM Tris–HCl buffer (pH 7.0) at 4 °C for 72 h to remove the ammonium sulphate.

#### DEAE-Sepharose Fast Flow chromatography

The sample was concentrated by polyethylene-glycol 20,000 (PEG-20M) at 4 °C, and the concentrated sample was loaded on DEAE-Sepharose Fast Flow chromatography (30 × 1.6 cm) and washed under the following conditions: Buffer A (50 mM Tris–HCl, pH 7.0); Buffer B (50 mM Tris–HCl, 2 M NaCl pH 7.0), with a flow rate of 0.5 mL/min. The sample was separated by use of the gradient elution method, and the protein elution surge was collected and assayed for protease activity and protein content. Active fractions were loaded by sodium dodecyl sulphate polyacrylamide gel electrophoresis (SDS-PAGE) and checked for their homogeneity.

#### Sodium dodecyl sulphate polyacrylamide gel electrophoresis

The molecular mass of purified protease was determined by SDS-PAGE, with its activity demonstrated to be 12% of Native-PAGE. Denaturing gel was stained in Coomassie Brilliant Blue R-250 for clear visualization of the bands. For zymogram analysis, the enzyme samples were separated using regular SDS-PAGE containing 0.2% casein in the separating gel. The SDS was omitted in the buffer and samples were not boiled. After each run, SDS from the gel was removed by incubating the gel twice in 2.5% Triton X-100 for 1 h. The gel was washed thrice for 20 min in distilled water to remove excess Triton X-100 and then incubated for 3 h in 50 mM Tris–HCl buffer (pH 7.0) at 37 °C. Finally, the gel was stained with a Coomassie Brilliant Blue R-250 staining solution.

### Comparison of characteristics between the purified protease and commercial neutral protease

#### Effect of pH and temperature on the activity and stability of protease

The different pH levels of the casein solution were used as the reaction substrate, in which pH 3.0-5.0 was prepared with a Na_2_HPO_4_-citric acid buffer solution, pH 6.0–8.0 was prepared with a Na_2_HPO_4_–NaH_2_PO_4_ buffer solution, and pH 9.0–10.0 was prepared with a NaOH–glycine buffer solution. The activities of the purified protease in the different pH values of the casein solution were measured respectively, and expressed as percentages of the highest activity, which was taken as 100%. The protease was incubated in a pH 3.0–10.0 buffer solution for 0–1 h at 4 °C, and the residual activity was measured every 10 min to determine the stability of the protease at different pH levels.

To determine the optimum temperature for the purified protease, its activity in 0.2 M sodium phosphate (pH 7.0) was measured at various temperatures (30–80 °C). The relative activities were expressed as percentages of the highest activity, which was taken as 100%. The protease was also incubated at various temperatures (30–80 °C) for 0–1 h, and the residual activity was determined every 10 min to determine the stability of the protease at different temperatures.

#### Effects of inhibitors and metal ions on protease activity

The inhibitors used were specific to serine proteases (phenylmethylsulfonyl fluoride, PMSF), cysteine proteases (iodoacetamide), aspartic proteases (pepstatin A) and metalloproteases (ethylenediaminetetraacetic acid, EDTA). Metal ions (NaCl, KCl, CaCl_2_, BaCl_2_, SnCl_2_, MgSO_4_, MnCl_2_, ZnCl_2_, CuSO_4_, FeSO_4_, Pb(CH_3_COO)_2_ and AlCl_3_) were added at a concentration of 0.2 mM each in order to determine their effects on the protease activity. In each case, the protease was incubated in the presence of the inhibitor/metal ion in 50 mM Tris–HCl buffer (pH 7.0) for 30 min at 4 °C before being assayed for the residual activity. The protease activity of control was defined as 100%.

### Kinetic parameters

The protease activities of the commercial protease and the purified protease were measured with different concentrations of casein, ranging from 0.5 to 3.0%, in 50 mM Tris–HCl (pH 7.0) at 40 °C, respectively. The Michaelis constant (*K*_m_) and the maximum rate (*V*_max_) of the enzyme-catalyzed reactions of these two proteases were computed with a Lineweaver–Burk plot.

### Data analysis

All the experiments were carried out in triplicate and the results expressed as mean values ± standard deviation. The data was subjected to an analysis of variance using statistics programming software (SPSS). A *p* value < 0.05 was considered to be statistically significant.

## Results

### Isolation and identification of strains with a high yield of protease

**Fig. 1:**
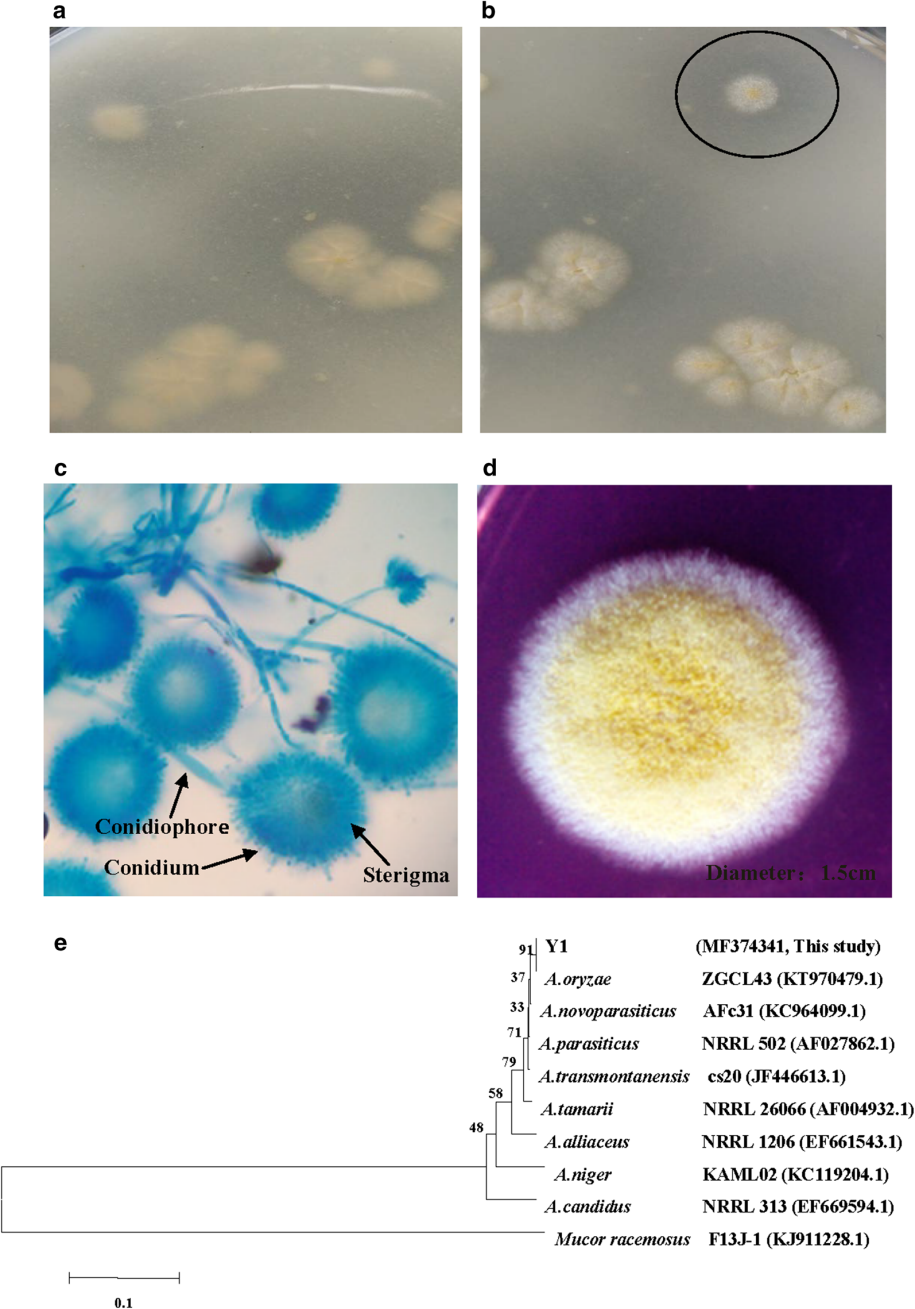
The hydrolysis circle of high protease-producing strain Y1 on the casein medium: **a** the back of plate; **b** the front of plate; **c** the colony morphology of strain Y1; **d** the results of strain Y1 by optical microscope; **e** phylogenetic tree showing the relationship between strain Y1 and other closely related fungi, with 0.005 substitution per-nucleotide

The strain Y1 was screened for its high capacity to hydrolyze protein from the samples. The strain has subsequently been preserved in the culture collection of the Sichuan Industrial Institute of Antibiotics, with the strain number siia9007. As shown in Fig. 1, the results revealed the strain Y1 to have a stronger ability to hydrolyze casein and there was an obvious hydrolysis ring on the casein plate. The colony morphology of strain Y1 is shown in Fig. 1c. The characteristics of strain Y1 included a 4–5 cm-diameter round shape, a neat edge, loose texture, and a color change from white to yellow. Figure 1d shows the morphological features of strain Y1. Through a comparative review of related reports (Samson et al. 2007; Varga et al. 2007; Houbraken et al. 2010), the strain Y1 was preliminarily identified as *Aspergillus* spp.

A phylogenetic tree (Fig. 1e) provides the relative positions of the strain Y1 as being closely related to *A. oryzae* (AF459735.1), with a similarity of 99%. The strain Y1 was, therefore, identified as *A. oryzae* (Fig. 1e).

### Enzyme extraction and purification

Purification results are summarized in Table 1. The specific activity of the purified protease increased from 226.9 to 2264.3 U/mg, while the protease was purified up to 10.0-fold with a recovery of 21% yield.

**Table 1:** Purification steps of neutral protease from *Aspergillus oryzae* Y1

Purification steps	Total activity (U)	Total protein (mg)	Specific activity (U/mg)	Purification (fold)	Recovery (%)
Crude enzyme solution	439,062	1934.7	226.9	1.0	100
(NH_4_)_2_SO_4_ (70%)	316,124	383.9	823.4	3.6	72.0
DEAEsepharose-FF	92,389	40.8	2264.3	10.0	21.0

**Fig. 2:**
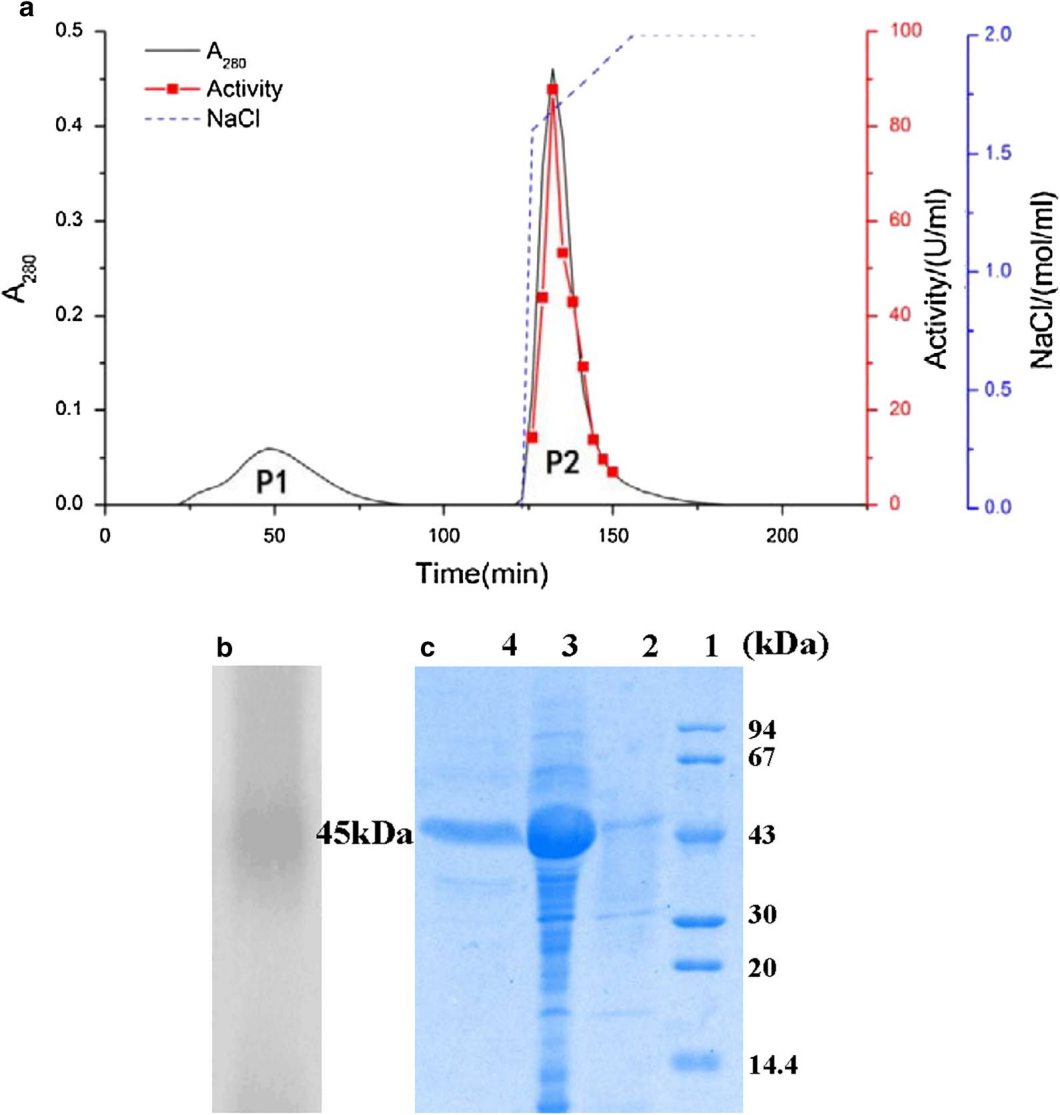
**a** Ion exchange chromatography of DEAE-Sepharose FF (30 × 1.6 cm, i.d); **b** zymogram of neutral protease activity; **c** SDS-PAGE analysis of crude and purified protease from *Aspergillus oryzae* Y1. Lane 1 low molecular weight; lane 2 crude extract; lane 3 ammonium sulfate precipitation; lane 4 purified neutral protease

The total activity and total protein of the crude enzyme showed a notably high specific activity of 439062U and 1934.7 mg, which is markedly higher than the enzyme activities previously reported in *A. oryzae* CH93 (14.7 U/g) (Salihi et al. 2016) and *Aeromonas veronii* PG01 (18.5 U) (Divakar et al. 2010). The crude enzyme was initially concentrated from 20 to 90% using ammonium sulfate. The dialyzed sample was separated by ion exchange chromatography, the chromatographic profile of which is shown in Fig. 2a, where P1 is a heterozygous protein peak and P2 is the peak of the target protease. The purity of the P2 was confirmed by a single band after Coomassie Brilliant Blue staining on 12% SDS-PAGE; the molecular mass was estimated at around 45 kDa (Fig. 2c). The proteolytic nature of the enzyme was confirmed by casein zymography, in which digested casein appeared as a white band corresponding to the protease position in the gel (Fig. 2b). The purified protease showed a single protein band on the 12% Coomassie Brilliant Blue R-250 stained gel.

### Effect of pH and temperature on the activity and stability of protease

**Fig. 3:**
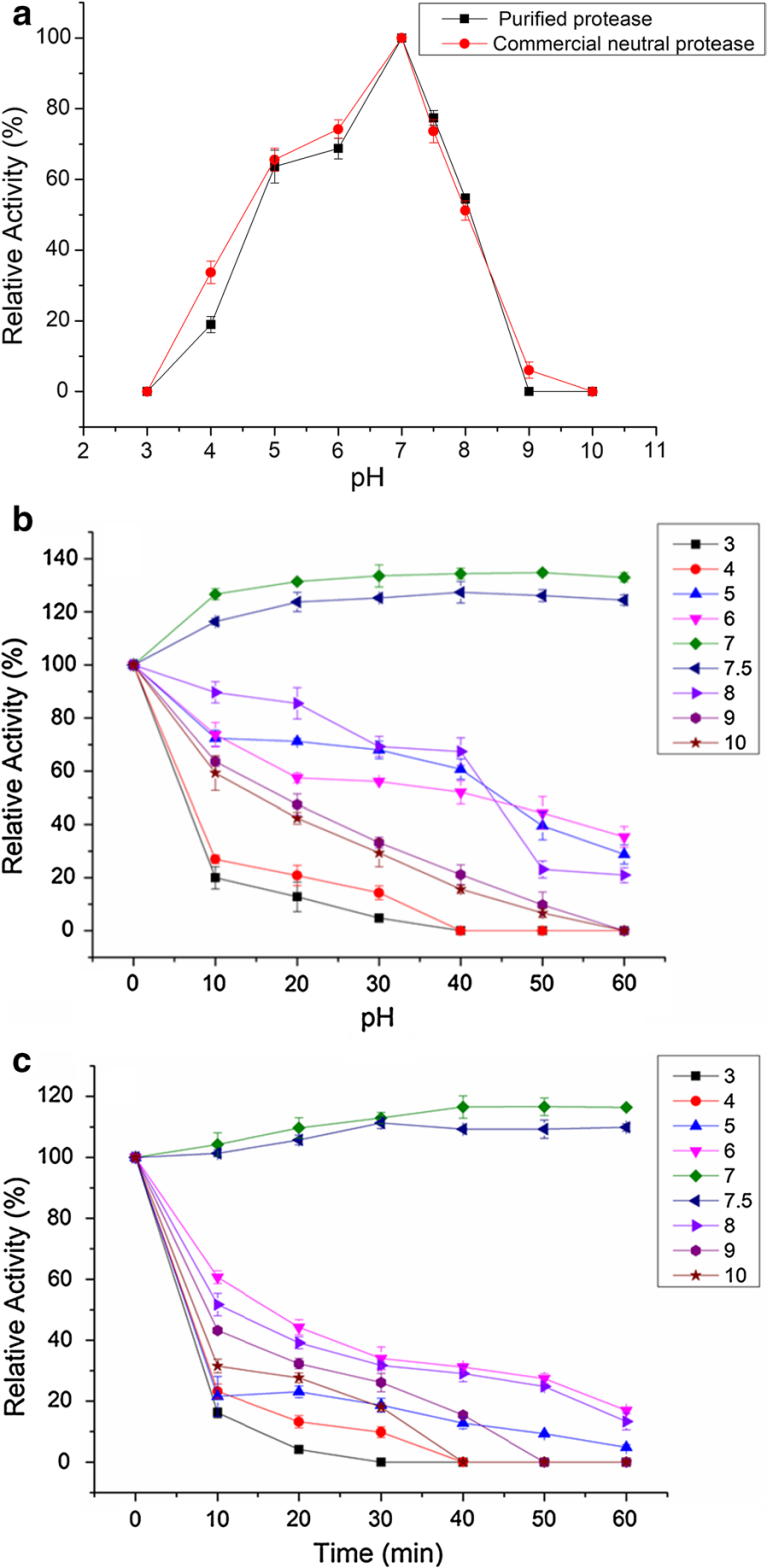
**a** Effect of pH on protease activity; **b** stability of purified protease at different pH, at 4 °C; **c** stability of commercial neutral protease at different pH, at 4 °C

The effect of pH value on the activity of the purified protease from *A. oryzae* Y1 and the commercial neutral protease is shown in Fig. 3a. The results indicate that the activity of both the purified protease and the commercial neutral protease increased from pH 3.0 to 7.0, and reached a maximum 61.75 and 48.23 U/mL at pH 7.0, thus suggesting that the purified protease was a neutral protease. The activity of the two kinds of protease showed good stability under neutral conditions at 4 °C, and the acid–alkali tolerance of the purified protease was stronger than that of the commercial protease (Fig. 3b, c).

**Fig. 4:**
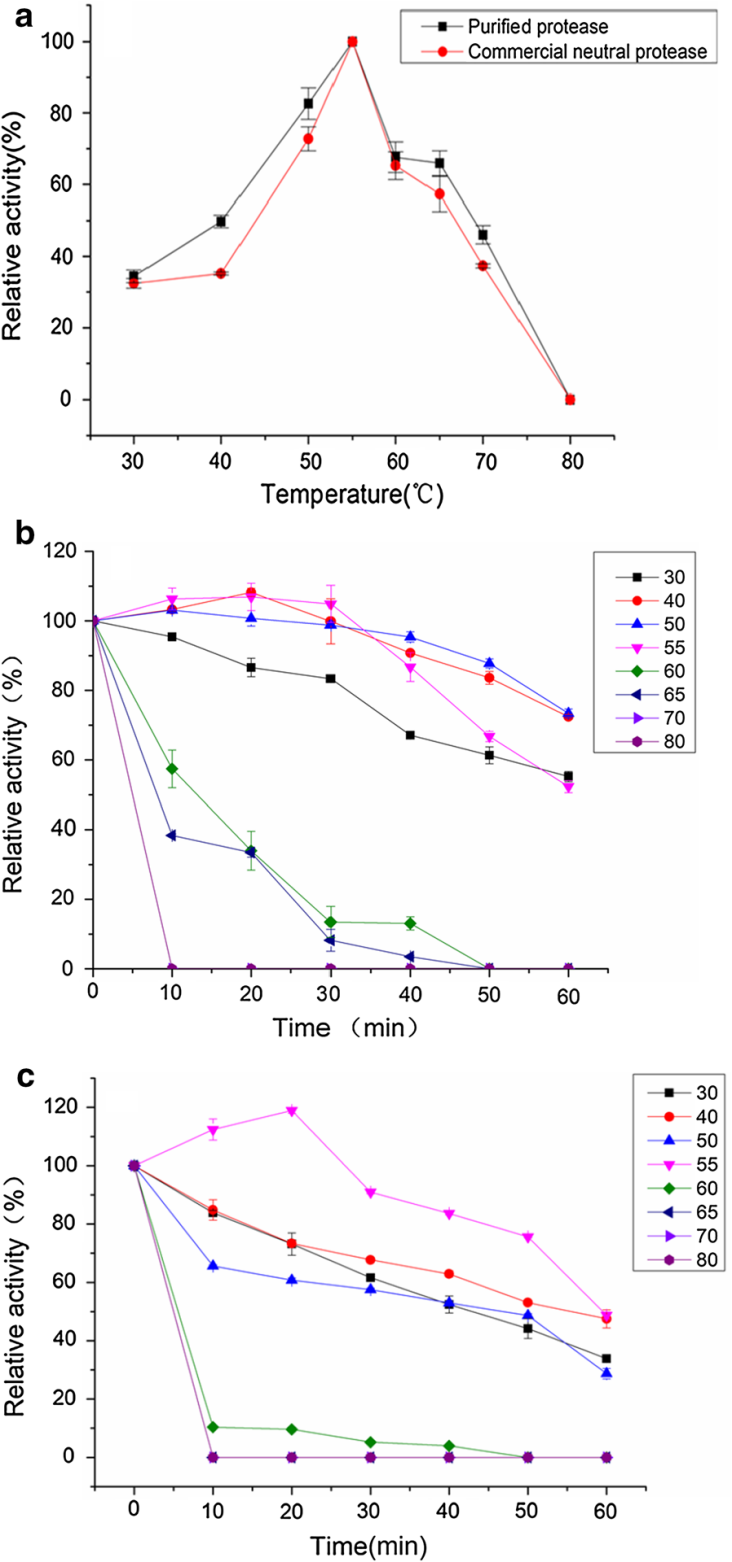
**a** Effect of temperature on protease activity; **b** stability of temperature on purified protease activity was determined at different incubation times (0–60 min) at different temperatures at pH 7.0. The residual protease activity was estimated under the enzyme assay conditions and indicated as the percentage relative to the activity of the untreated protease activity; **c** stability of temperature on commercial neutral protease activity was determined at different incubation times (0–60 min) at different temperatures at pH 7.0. The residual protease activity was estimated under the enzyme assay conditions and indicated as the percentage relative to the activity of the untreated protease activity

The effect of temperature on the purified protease and the commercial neutral protease is shown in Fig. 4a, in which the results indicate that the activity of both increased from 30 to 55 °C, and reached a maximum 102.38 and 97.10 U/mL at 55 °C. The activity of the purified protease stabilized at 50 °C for 60 min (Fig. 4b), while the activity of the commercial neutral protease remained stable at 40 °C for 30 min (Fig. 4c). This indicates that the thermal stability of the purified protease is better than that of commercial neutral protease.

### Kinetic parameters

**Fig. 5:**
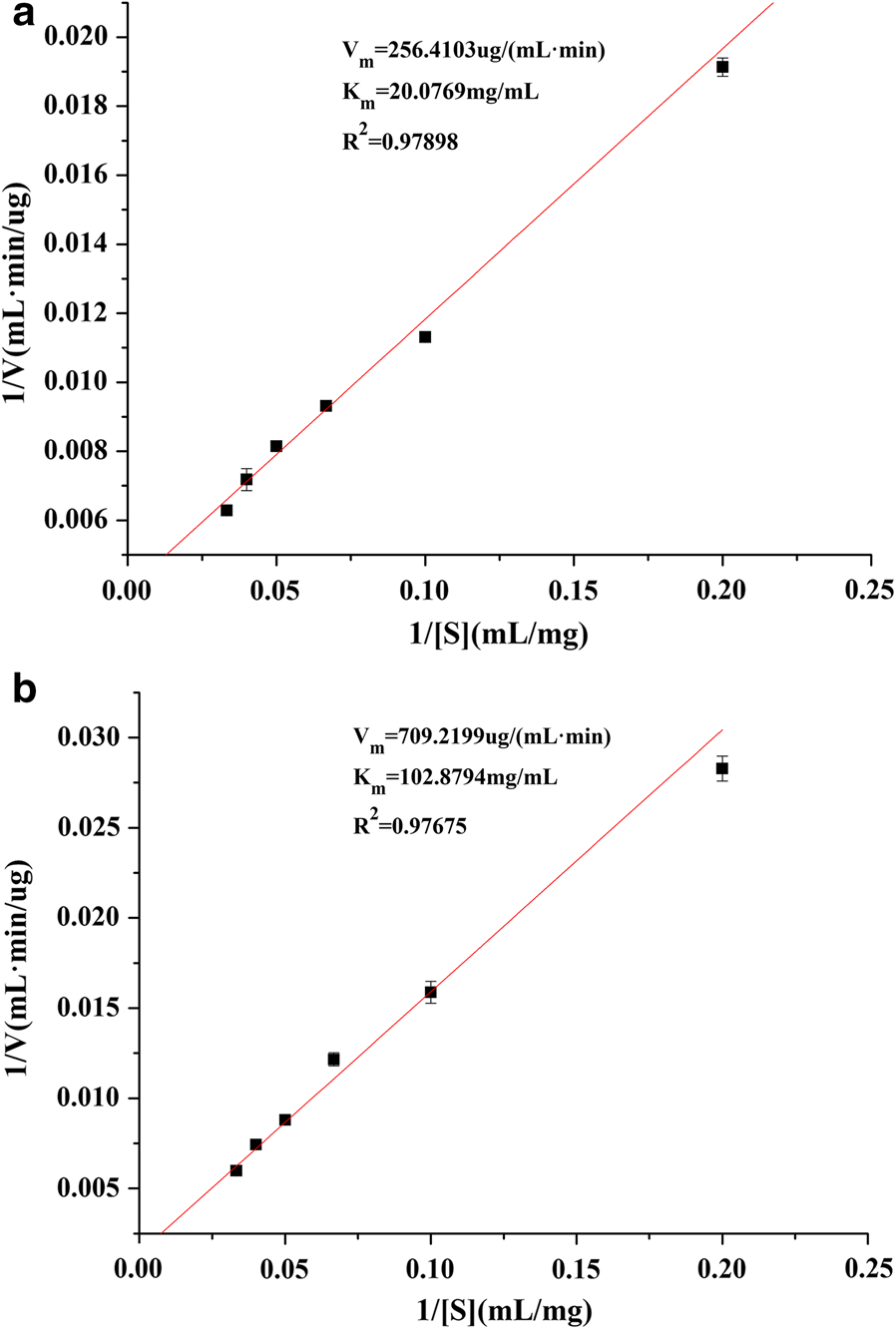
The Lineweaver–Burk plot of the neutral protease from *Aspergillus oryzae* Y1 (**a**) and the commercial neutral protease (**b**)

The catalytic activity of protease was determined at 55 °C and pH 7.0 with varying casein concentrations ranging from 5.0 to 30.0 g/L. The results of the purified protease were plotted according to Lineweaver–Burk, as shown in Fig. 5a, with the *K*_m_ and *V*_max_ at 20.0769 mg/mL and 256.4103 g/mL min, respectively. The values of *K*_m_ and *V*_max_ for the commercial neutral protease were 102.8794 mg/mL and 709.2199 μg/mL min, respectively (Fig. 5b). When the substrate and *V*_max_ were the same, the *K*_m_ of the purified protease was much smaller than that of the commercial neutral protease, thus indicating that the purified protease had a higher affinity for the substrate than that of the commercial neutral protease.

### Effects of inhibitors and metal ions

The effects of different inhibitors on the activity of the purified protease and the commercial neutral protease were determined by pre-incubating 1.0 mM of the inhibitors at 4 °C for 30 min (Table 2). The activities of both proteases were strongly inhibited by 1 mM EDTA and PMSF. In addition, 53% of the initial activity of the purified protease was inhibited by IAM. Aspartic protease inhibitors and Pepstatin A showed a moderate effect on the purified protease after 30 min incubation at 4 °C, whereas IAM and Pepstatin A demonstrated a slightly inhibitive effect on the activity of the commercial neutral protease.

**Table 2:** Effect of different inhibitors and metal ions on the protease activity

Reagent	Concentration (mM)	Relative activity of protease (%)	
		Purified protease	Commercial neutral protease
Control		100	100
PMSF	1.0	27.89 ± 1.55	49.8 ± 2.77
EDTA	1.0	18.85 ± 0.95	34.99 ± 0.06
IAM	1.0	47.03 ± 1.53	92.31 ± 0.21
Pepstatin A	1.0	63.04 ± 2.85	92.33 ± 1.15
KCl	0.2	158.56 ± 3.45	141.17 ± 1.18
NaCl	0.2	115.92 ± 3.67	112.69 ± 1.32
CaCl_2_	0.2	273.44 ± 3.56	246.02 ± 4.44
SnCl_2_·2H_2_O	0.2	174.29 ± 2.56	162.56 ± 3.63
CuSO_4_·5H_2_O	0.2	325.99 ± 4.51	303.33 ± 3.29
MgCl_2_	0.2	57.67 ± 3.44	49.58 ± 3.62
BaCl_2_·2H_2_O	0.2	42.13 ± 1.65	30.17 ± 1.42
MnCl_2_·4H_2_O	0.2	244.76 ± 3.78	233.85 ± 2.28
ZnCl_2_	0.2	91.39 ± 3.76	88.66 ± 4.08
Pb(CH_3_COO)_2_·3H_2_O	0.2	123.69 ± 3.84	118.47 ± 4.03
FeSO_4_·7H_2_O	0.2	124.76 ± 2.85	122.62 ± 4.7
AlCl_3_	0.2	153.93 ± 4.77	150.6 ± 2.96

The purified protease activity from *Aspergillus oryzae* Y1 and commercial neutral protease activity without the addition of metal ions was defined as 100%

The effects of different metal ions on the protease activity are presented in Table 2. The activity of the purified protease was enhanced up to 173 and 145% by Ca^2+^ and Mn^2+^, respectively; the activity of the purified protease was increased by metal ions Na^+^, Sn^2+^, Cu^2+^, Pb^2+^, Fe^2+^ and Al^3+^, with the proportions of increase as 16, 74, 49, 48, 24, 25 and 54%, respectively. Activity was inhibited by Mg^2+^, Ba^2+^ and Zn^2+^ in varying degrees in comparison with the control. The effect of metal ions on the activity of purified protease was roughly coincident with that of commercial neutral protease.

## Discussion

A number of comparative benefits of purified protease from *A. oryzae* Y1 have been ascertained in this study. For one, the molecular mass of purified protease from Y1 was found to be inconsistent, unlike that of other neutral proteases, with a molecular mass of 43 kDa, which is larger than those found in *Bacillus megaterium* (25, 28 kDa) (Asker et al. 2013), *Aeromonas veronii* PG01 (33 kDa) (Divakar et al. 2010), *Bacillus* (34 kDa) (Breite et al. 2010), and *A. parasiticus* (36 kDa) (Anitha and Palanivelu 2013). The activity of the purified protease was strongly inhibited by 1 mM EDTA and PMSF, and, thus, was identified as a metal-dependent serine protease. A number of previous studies have been conducted on serine protease with molecular masses of approximately 25–37 kDa (Asker et al. 2013; Qiuhong et al. 2006; Cavello et al. 2013), all of which show the proteases concerned to be independent of metal ions. These results, therefore, suggest that the purified protease from *A. oryzae* Y1 is indeed a distinct protease.

The activity of the purified protease demonstrated a wide range of pH 4.0–8.0, and reached its highest value in pH 7.0. The purified protease was, therefore, identified as a neutral protease, which is consistent with the protease from *A. flavipes* and *A. brasiliensis* (Novelli et al. 2016). Furthermore, compared with the commercial neutral protease, this purified protease demonstrated better stability under the conditions of weak acid and weak alkalinity. It was further confirmed that the protease meets the basic requirements of the food industry. In particular, the protease’s activity was improved by Na^+^ in the buffer at 4 °C and then tended to stabilize, as can be seen in Fig. 3b. Sodium chloride is one of the main ingredients in fermented broad beans, and the promotion of sodium ions will help to increase the enzyme activity during the production of broad-bean sauce. Additionally, the optimum temperature of the purified protease was found to be 55 °C, which is consistent with previous reports (Vishwanatha et al. 2009; Ma et al. 2016) and, in comparison with the commercial neutral protease and the neutral protease from *A. oryzae* HDF-7 (Yu et al. 2011), the thermal stability of the purified protease proved to be clearly superior. This is contrary to previous assumptions about neutral proteases, which generalized them as mesophilic and is intolerant of high temperatures (Huang et al. 2013). Few previous reports have recorded thermostability in the neutral protease from *A. oryzae*.

In the presence of some metals, the activity of the purified protease was varied. Activity was enhanced to 173 and 145% by 0.2 mM Ca^2+^ and Mn^2+^, respectively, concurring with the findings of previous studies (Kamran et al. 2015; Yadav et al. 2015). Ca^2+^ played a significant role in enhancing the activity of most proteases, possibly due to the stabilization of the enzyme in its active conformation, as opposed to its being impacted in the catalytic reaction (Strongin et al. 1978). It was found that two calcium binding sites at the serine protease active site, along with the thermal stability of the protease, decreased activity significantly by removing a calcium ion from the strong binding site (Lee and Jang 2001). Additionally, Mn^2+^ has been noted to bind to the amino acid residues of the protease side chains and stabilize their conformation without changing the conformation of the protease catalytic site. Yadav et al. (2015) reported that protease activity is increased by Na^2+^ (140%) and Fe^2+^ (156%) at 2 mM; and, furthermore, that Sn^2+^, Cu^2+^, Pb^2+^ and Al^3+^ exert stimulating effects at 0.2 mM concentration, however there are few corroborating reports of these findings. In most studies, Cu^2+^ has been reported as a strong inhibitor; for example, when adding 1–5 mM Cu^2+^, the protease activity decreases by between 12% to 98% (Divakar et al. 2010; Yu et al. 2011; Yadav et al. 2015; Ferrareze et al. 2016; Abidi et al. 2011; Hsiao et al. 2014; Khaled et al. 2011). Protease inhibition by Mg^2+^, Ba^2+^ and Zn^2+^ were also reported by Norifumi et al. (2012), who suggested that these metal ions chemically react with the protein group in the polypeptide chain of the enzyme, resulting in the loss of protease activity (Kamran et al. 2015).

In most of previous studies, the tested concentration of metal ions acting on proteases was generally 1.0–10.0 mM, however, in this study the concentration was 0.2 mM. The pre-experiment confirmed that the purified protease activity was markedly stimulated at 0.2 mM. Moreover, it was found that a low concentration of Cu^2+^ could shorten the maturation time of *A. oryzae* Y1 and, consequently, increase the yield. Overall, the findings resulting from this work should prove to be beneficial to the production of *Aspergillus oryzae*, not only for pea sauce production but in various fields of the food processing industry.
